# Relevance of Sociodemographics and Clinical Tests in Single- and Dual-Task Conditions as Gait Speed Predictors of Parkinson’s Disease

**DOI:** 10.3390/jcm11030757

**Published:** 2022-01-30

**Authors:** Constanza San Martín Valenzuela, Lirios Dueñas, José M. Tomás, Patricia Correa-Ghisays, Pilar Serra-Añó

**Affiliations:** 1 Unit of Personal Autonomy, Dependency and Mental Disorder Assessment, Faculty of Medicine, University of Valencia, 46010 Valencia, Spain; constanza.martin@uv.es (C.S.M.V.); patricia.correa@uv.es (P.C.-G.); 2 Department of Physiotherapy, Faculty of Physiotherapy, University of Valencia, 46010 Valencia, Spain; pilar.serra@uv.es; 3 UBIC Research Group, Department of Physiotherapy, Faculty of Physiotherapy, University of Valencia, 46010 Valencia, Spain; 4 Centro Investigación Biomédica en Red de Salud Mental, CIBERSAM, 28029 Madrid, Spain; 5 Physiotherapy in Motion, Multi-Speciality Research Group (PTinMOTION), Department of Physiotherapy, Faculty of Physiotherapy, University of Valencia, 46010 Valencia, Spain; 6 Department of Behavioral Sciences Methodology, Faculty of Psychology, University of Valencia, 46010 Valencia, Spain; Jose.M.Tomas@uv.es

**Keywords:** dual-task, gait rehabilitation, gait speed, Parkinson’s disease, functional assessment

## Abstract

This cross-sectional study aimed to identify the patient characteristics and clinical test results that predict the functional gait speed of people with Parkinson’s disease (PD). The impact of dual tasks on gait in Parkinson’s disease (PD) reveals a lack of automaticity and increased cognitive demands. We explored which characteristics explained walking speed with and without dual-task interference and if they reflected the cognitive demands of the task. The preferred gait speed, stride length, and cadence were measured in individuals with PD through five conditions: single-task (ST) and visual, verbal, auditory, and motor dual-tasks (DTs). Sociodemographic and disease characteristics and the results from clinical tests such as the Dynamic Parkinson’s Disease Gait Scale (DYPAGS), Frontal Assessment Battery (FAB), and Parkinson’s Disease Questionnaire-39 (PDQ-39), among others, were also recorded. Two models of multiple regression analysis were used to explore the predictive value of outcomes concerning speed. In Model I, clinical results were included, and in Model II, spatiotemporal variables were added to the significant predictors of Model I. Forty PD patients (aged 66.72 (7.5) years) completed the assessments. All the models generated were significant (*p* < 0.01). Models I and II accounted for 47% and 93% of the variance, respectively, in the single-task condition. A patient’s gender, prescribed medication (drugs), academic level, and Hoehn and Yahr (H&Y) stage, along with the FAB, DYPAGS, and PDQ-39 scores, were significant predictors of gait speed in Model I for the ST and DT conditions. In Model II, the H&Y stage and prescribed medication (drugs), along with the FAB and DYPAGS scores, remained significant predictors. This research found that sociodemographics, the patient’s stage disease, and their clinical test results contribute to their walking speed, highlighting the multifactorial nature of gait in demanding environments.

## 1. Introduction

Parkinson’s disease (PD) is a chronic, progressive, and neurodegenerative pathology, with motor and non-motor disorders [1], which affects functional mobility [2]. In turn, people with PD report that gait impairments are the most disabling motor symptoms of the disease [3]. Indeed, axial symptoms in PD such as disturbances in gait and posture are often resistant to drug treatment, deteriorate as the disease progresses, increase the likelihood of falls, and result in higher rates of hospitalization and mortality, thus having a negative impact on the patients’ quality of life [4,5]. For this reason, several studies have characterized the gait of people with PD, observing the distinctive deterioration of step and stride length (−12.60%), cadence impairment (−8.48%), and the subsequent alteration of gait speed (−22.3%) and double support time increase (12.38%) [6,7,8]. Of particular interest is the speed of the gait, since it is considered a key indicator of mobility and has been linked to independence, community engagement, morbidity, and mortality [9]. In fact, gait speed in older adults has been accepted as a reliable and sensitive measure of physiological performance and prediction of clinical outcomes, to diagnose frailty [10], and has indeed been regarded as the “sixth vital sign” [11]. In PD, slow speed is related to clinically advanced disease, poor mobility, and higher falling risk, among others [12]. In this sense, different studies have shown a correlation of gait speed with variables from other domains than the physical one, such as cognitive decline [13,14,15,16]—which becomes more evident as PD progresses and has a significant impact on its long-term prognosis [17]—or the clinical and nutritional domains [18]. Going further, a slow gait is considered a risk factor for suffering from severe COVID-19, possibly leading to death [19].

However, gait speed recording could undervalue the complexity of some neurologic chronic pathologies like PD. In this event, the functional assessment of gait has become a methodology widely used by researchers. This is known as dual-tasking and involves the development of two tasks with different objectives at the same time [20], where attention is afforded to one of the tasks or alternates between the primary task (gait) and the secondary task (i.e., cognitive or motor). In the PD population, performing secondary tasks while walking further deteriorates the gait and restricts how much walking can be achieved in functional contexts of daily life [6]. Gait assessment with secondary functional tasks, such as talking with another person or carrying something with the hands, allows researchers to observe the performance of people with PD in everyday contexts and guides physical therapy with the aim of preparing patients for probable challenges they will face. This is of special interest in PD because the basal ganglia are involved in the control of different aspects besides motor control [21,22], and the onset of cognitive impairment can further impair the performance of daily dual-tasks (DTs). Gait problems related to impaired dual-tasking capacity are frequent in the PD population and have been demonstrated in previous works [8,17,23].

Photogrammetry is considered the gold standard for gait assessment. However, this evaluation system requires individuals to undergo motion analysis, which may not be practical in a clinical setting. There are other “clinic-friendly” assessments that can also provide a robust examination of the gait and physical function and that can predict some outcomes related to the quality of the gait. As such, it is common in clinics to use scales and tests that allow for quick administration and that have zero cost.

In this context, the main objective of the study was to analyze whether sociodemographics and clinical test outcomes could predict the gait speed in single- and dual-task conditions. The secondary aim was to learn which variables might determine gait speed when analyzed together with biomechanical parameters as control predictors.

## 2. Materials and Methods

### 2.1. Study Design and Setting

The study design was cross-sectional with a convenience sample (specifically, modal instance sampling) and adhered to the STROBE guidelines (http://www.strobe-statement.org/ accessed on 9 October 2021). The data in this paper originate from pre-intervention measurements in a single-blind randomized control trial (RCT) study, with eight weeks’ follow-up and two parallel-groups (1:1), conducted in Valencia, Spain [24] from June 2014 to June 2016. The study was registered under number NCT04038866 at clinicaltrial.gov and the Ethics Committee of the University of Valencia approved all the procedures that were performed (no. H1397723257189).

### 2.2. Participants

Participants were recruited from two centers: a Parkinson’s disease association and the neurology service of a local public hospital. The sample characteristics have been described in detail previously [8]. Eligible participants were all adults previously diagnosed with idiopathic Parkinson’s disease, who met the following inclusion criteria: (1) Hoehn and Yahr (H&Y) stages I, II, or III; (2) two months prior to the study without any physical or cognitive training; (3) able to walk independently; (4) held a normal cognitive state (i.e., score > 25) according to the Mini-Mental test adapted for PD [25]; and (5) with symmetry in the lower limb lengths (<1 cm). Exclusion criteria were (1) the presence of another neurological or symptomatic musculoskeletal disease (e.g., joint pain due to arthritis); (2) history of trauma or surgery on the lower limbs; (3) balance disorders related to other pathologies or history of falls during the year before to the study participation; (4) uncontrolled chronic diseases (e.g., hypertension or diabetes); (5) changes in the levodopa treatment during the development of the study, and (6) impairment cognitive state and/or depression.

### 2.3. Outcomes and Measurement Procedures

The assessment sessions were performed at the Medicine Department of the University of Valencia in the on-medication state [6] and included, first, a clinical interview to record the main personal data of the participants and to verify their cognitive state (measured by a psychologist); second, an anthropometric evaluation to record their weight, height, and lower limbs’ lengths [26], which were used to standardize the biomechanical variables; third, a biomechanical assessment of the gait; and last, clinical and psychometric assessment through different questionnaires and tests. The demographic and disease-related data recorded were (i) Age; (ii) Gender; (iii) BMI; (iv) Academic level; (v) Number of comorbidities; (vi) Number of prescribed medications (drugs) taken; and (vii) H&Y stage of disease severity.

#### 2.3.1. Gait Assessment

All the participants walked barefoot [8] at a self-selected comfortable speed under all conditions tested. Five conditions were randomly evaluated: (1) single-task (ST)—walking without secondary tasks with the attention focused only on walking performance; (2) visual DT—walking while checking the time on an analog clock projected at the end of the walkway; (3) verbal DT—walking while telling the evaluator the activities they had performed the previous day in chronological order; (4) auditory DT—walking while listening to and recognizing different daily noises; and (5) motor DT—walking while carrying one glass in each hand and repeatedly transferring their contents (beans) from one to the other. Respectively, these last four tasks were intended to simulate the action of looking at the time, talking to another person, listening to the sounds of the environment, and manipulating objects with the hands while walking. All of these tasks are representatives of daily life activities, which provide external validity to the study and concur with skills previously studied [6,27]. During the DT conditions, participants were urged to focus their attention on the secondary task all the time they were walking.

Gait assessment was carried out in a corridor 10-m long, and data were registered using 3D photogrammetry with 12 smart cams (Kinescan/IBV software, Biomechanical Institute of Valencia, Valencia, Spain, version 5.3.0.1) and two force platforms (Dinascan/IBV Biomechanical Institute of Valencia, Valencia, Spain). A valid repetition was one in which the patient performed at least five complete strides along the corridor and where one complete and isolated footprint (right or left) from the third stride coincided with the dynamometric platform located in the center of the corridor. The previous and subsequent strides were discarded to avoid acceleration and deceleration phases, at the beginning and end of the gait, respectively. For each of the five evaluated walking conditions, 10 repetitions were performed—five with each foot—so that we could later use the average of these. Participants were allowed to rest between repetitions, sitting on a stool for less than three minutes, when they reported fatigue.

The biomechanical model was composed of 35 landmarks at specific anatomical points on both sides of the body [8], which represented the trunk, pelvis, thigh, leg, and foot segments. Before recording their gait, participants were allowed to walk into the corridor (ST condition) to familiarize themselves with the test. For the calculation of biomechanical variables based on the data exported from the photogrammetry system and the dynamometric platform, the software was programmed with MATLAB (MathWorks, Natick, MA, USA, version R2016b). The average of these dependent variables was calculated from the 10 repetitions for each condition: (i) Gait speed—distance traveled by the body per unit of time (m/s); (ii) Stride length—distance measured between two consecutive heel strikes of the same foot (m); and (iii) Cadence—number of steps taken in a minute (steps/min).

#### 2.3.2. Clinical and Psychometric Assessment

- Quality of life

The Parkinson’s Disease Questionnaire-39 (PDQ-39) is a Parkinson’s disease-specific instrument for the evaluation of the quality of life [28]. This questionnaire consists of 39 items covering eight dimensions: mobility (10 items), activities of daily living (6 items), emotional wellbeing (6 items), stigma (4 items), social support (3 items), cognitions (4 items), communication (3 items), and bodily discomfort (3 items). Each dimension is scored individually and has a score range of 0 (never have difficulty) to 100 (always have difficulty). The higher the score, the poorer the quality of life. The items evaluate the condition of the individual during the last month, and although it is usually the patient who fills out the survey, a researcher helped the subjects with the questionnaire’s completion to ensure their comprehensive understanding [29]. High reliability and good validity of the PDQ-39 have been previously determined for the Spanish version, as was used for the present study [30].

- Executive cognitive functions

The Frontal Assessment Battery (FAB) is a screening test that assesses deficits in executive functions, and the Spanish version of this was used in the current study [31]. The FAB consists of six subtests that explore the cognitive control processes responsible for appropriate goal-directed behavior and response to challenging situations. Each subtest is scored from 0–3, with a total maximum score of 18 indicating top performance on the test, and thus, better functioning [32]. The FAB is a reliable and valid scale [31,32] and has been extensively used in research on people with PD [15,16]. Additionally, the Trail Making Test (part A (TMTa) and B (TMTb)) was applied, a widely used paper-and-pencil neuropsychological instrument measuring the executive function [33]. The test consists of two parts: during the TMTa, subjects are instructed to connect encircled numbers from 1 to 25 in ascending order, which are randomly distributed on a piece of paper. The TMTb consists of connecting encircled numbers (1 to 13) and letters (A to L) in alternating order (1–A–2–B…). Performance on this test is assessed by scoring an individual’s completion time. Both parts of the TMT reflect the visual search function and motor speed, while the TMTb also captures set shifting, attention switching, simultaneous maintenance of two sequences, and the working memory. The Trail Making Test is a reliable [34] and valid tool [35] when assessing an adult population, and has been broadly used in research on people with PD [36,37].

- Physical and gait clinical performance

The Dynamic Parkinson’s Gait Scale (DYPAGS) [38] was used for clinical gait assessment and is comprised of eight relevant items for the objective quantification of PD gait features: walking forward/backward/in a dual-task, turning to both sides, imaginary obstacle avoidance with both legs, and passing through narrow spaces. Each of the items has a score from 0 to 5 (assigned according to the performance achieved), with a total score of 40 points. High scores represent severe gait disorders related to PD. The DYPAGS is a reliable and valid scale [38] commonly used to assess the gait performance of individuals with PD. Additionally, the Tinetti Performance-Oriented Mobility Assessment (Tinetti) was used to measure gait and balance. The Tinetti is a valid and reliable measure of mobility in the elderly [39] and those with PD [40]. The Tinetti scale is comprised of 16 items, divided into two components to assess the gait and balance functions independently. The maximum score for balance is 16 and for gait is 12, adding up to a total of 28 points. Higher scores indicate better performance. Lastly, the Timed Up and Go test (TUG) [41] was used to assess the degree of balance and the functionality of individuals with PD [42]. The test consists of getting up from a chair, walking 3 m, turning around, returning to the chair, and sitting down. The time to complete the task is measured with a stopwatch. A modified version adopted by various authors, in which subjects are asked to walk as fast as they can, was included in this study [43]. The use of the TUG in PD patients has good reliability and validity [44].

### 2.4. Data Analyses

Statistical analyses were performed using IBM SPSS v.24 (SPSS Inc., Chicago, IL, USA). Descriptive statistics, including means and standard deviations, were calculated to assess characteristics including age, body mass index, and other demographic information. As the assumption of normality was fulfilled in the variables analyzed and residuals, a stepwise multiple linear regression analysis model was used with the predictive power on gait speed of several predictor variables [45]. Stepwise regression was employed given the large number of predictors relative to the number of participants. Stepwise regression allows one to obtain a regression model only with the minimum number of statistically significant predictors [46].

Two multiple linear regression models were performed for the speed measured in each gait condition. To achieve the main aim of this study, the first model (i.e., Model I) included as predictors the sociodemographic and disease characteristics and the results of the aforementioned clinical tests. To respond to the secondary objective, the second model (i.e., Model II) included significant predictors of Model I, along with the stride length and cadence as determinants of speed in themselves. By doing this, it was verified which clinical variables continue to predict speed, even when they share a regression model with stride and cadence.

## 3. Results

A total of 40 participants completed the study, with a mean age of 66.72 (7.59) years. The participants’ characteristics and outcome measures are presented in Table 1 and Table 2, respectively. Regarding the analysis of Model I, a statistically significant regression model was found for speed in the single-task condition (F_(3,33)_ = 9.75, *p* < 0.01) and the visual (F_(2,34)_ = 5.41, *p* < 0.01), verbal (F_(1,35)_ = 4.33, *p* < 0.05), auditory (F_(2,34)_ = 5.92, *p* < 0.01), and motor (F_(3,33)_ = 8.44, *p* < 0.01) dual-task conditions. The single-task speed model and motor dual-task speed model accounted for 47% and 43% of the variance, respectively. The models that predicted speed in the dual visual, verbal, and auditory cognitive tasks explained 24%, 11%, and 25% of the variance, respectively. Of the models generated, the FAB test (SBeta = 0.54, *p* < 0.01 in single-task) and DYPAGS scale (SBeta = −0.46, *p* < 0.01 in motor dual-task) were the most important clinical variables to predict the speed in the models of the different gait conditions. On the other hand, the gender (SBeta = 0.36, *p* < 0.01 in single-task), prescribed medication (drugs) taken (SBeta = −0.32, *p* < 0.05 in visual dual-task), academic level (SBeta = 0.33, *p* < 0.05 in verbal dual-task), and H&Y stage (SBeta = 0.33, *p* < 0.05 in auditory dual-task) were the only significant characteristics in the models. Table 3 shows all the predictors and coefficients for Model I through the conditions.

### Formatting of Mathematical Components

Regarding the analysis of Model II, a statistically significant regression model was also found for gait speed in the single-task condition (F_(3,36)_ = 165.14, *p* < 0.01) and visual (F_(2,34)_ = 5.41, *p* < 0.01), verbal (F_(2,35)_ = 307.49, *p* < 0.01), auditory (F_(4,35)_ = 37.39, *p* < 0.01), and motor (F_(2,37)_ = 197.58, *p* < 0.01) dual-task conditions. The single-task speed model accounted for 93% of the variance, while in the visual, verbal, auditory, and motor dual-tasks conditions, the models explained 24%, 94%, 81%, and 91% of the variance, respectively. The speed in the visual condition remained the same in both models (I and II). When adding the stride length and cadence into the models, the FAB test (SBeta = 0.27, *p* < 0.01) and the H&Y stage (SBeta = −0.19, *p* = 0.01) were only significant in the speed model of the auditory condition. Table 4 shows all the predictors and coefficients for Model II throughout the conditions.

## 4. Discussion

The main objective of this study was to analyze sociodemographic and clinical tests as predictors of gait speed in different measurement conditions (i.e., without and with dual-task) for people with untrained Parkinson’s disease. To learn the relevance of those significant predictors (secondary aim), they were introduced into a second regression model with the spatiotemporal parameters that directly conditioned the gait speed (i.e., stride length and cadence). When only the clinical outcomes and participant characteristics were used as predictors, a significant model (i.e., Model I) was determined, accounting for 47% and 43% of the gait speed variance in the single-task and motor dual-task conditions, respectively. Meanwhile, the models that predict speed in the visual, verbal, and auditory dual cognitive tasks explained 24%, 11%, and 25% of the gait speed variance, respectively. Regarding regression Model II, the explained variance of gait speed in the different conditions was observed to be around 90%, except when walking while performing a visual task, for which the predictors were the same as those found in Model I.

Similar to our study, Shearin et al. [45] performed a regression analysis to predict the gait speed from clinical tests on PD patients, explaining 58% of the variability of gait speed in a single-task condition. Yet, these authors used clinical tests of the gait, cognition, and disease progression that differed from those used in our study, and the authors also included tests related to dynamic balance and plantar flexor strength, which could explain small differences in the variance of the models. Unlike Shearin et al.’s study, in our work, single-task gait speed was also conditioned by the gender of the participants, relating higher speeds to the male gender. The predictors of gait speed, while performing a motor task, were found to be similar to those of the single-task condition (i.e., DYPGS and FAB). This may be due to the fact that dual-motor task requires a similar motor control to the single-task walk [24,47]. In contrast, Model I in the cognitive dual-task conditions only explained up to 25% of the variance in gait speed, which may indicate that the gait speed of participants under cognitive interference depends on additional factors to those studied in this paper. Additionally, the predictors of the gait varied between different cognitive dual tasks, which could indicate that diverse neural resources condition the speed performance in environments with different requirements. More studies are warranted to elucidate this hypothesis/aspect.

Additionally, under cognitive interference, we observed an order of deterioration of the gait, with lower speeds when participants perform a verbal task [8,24]. This coincides with the variance that could be explained in each regression model, since in the model for the dual-task verbal condition, only an R^2^ of 0.11 was obtained, where the academic level of participants was the only predictor. Such a finding demonstrates that functional movement is complex, with multiple systems involved beyond the musculoskeletal one [48]. A low academic level has been linked to low maximum gait speeds [49], though mediated by factors such as a higher body mass index and lifetime exposure to a physical workload, which shows that the cognitive resources acquired over the course of life influence an individual’s motor control. Furthermore, in Model I of the gait speed with a visual task, it was observed that taking fewer prescribed medications (drugs) was related to a higher gait speed, which has previously been stated by other authors in healthy older people [50] and in a study on the impacts on the ability to walk for people with PD [51].

In Regression Model II, when introducing step length and cadence as predictors beside the significant variables found in Model I, it was observed that the explained variance of speed in different conditions was around 90%, except while performing a visual task (for which the predictors (i.e., prescribed medication (drugs) taken and DYPAGS) were the same as those in Model I). This can be explained by the suggestion that when the speed is disturbed by a visual task, the success of the gait itself depends on other factors related to the vision and the systems affected by it (e.g., balance), rather than only those related to the physical status, even when the visual task is not the most disruptive to walking fast. Yet, the level of interference from the secondary tasks used in the study might also have been influenced by individuals’ daily practices, as the tasks were similar to regular day-to-day tasks and, therefore, may not have caused equitable interference in the gait speed. On the other hand, the differences in variance explained by Models I and II lay mainly in the stride and cadence, which are variables that determine the individual’s speed. The relationship between cadence and stride length has been shown to be linear in a broad range of velocities in both healthy individuals and patients with neurological gait disorders [52]. Although a linear increase of cadence and stride length in patients with PD is parallel to healthy subjects, they show lower stride length at any given velocity [8,53,54]. Moreover, a model based on clinical tests has limitations associated with using qualitative questionnaire-based measurements [45]. In addition, other factors such as falls, freezing of gait, and anxiety may have accounted for the variability of speed, but were not assessed in this study.

In regression Model II, no significant clinical predictors remained when spatiotemporal parameters were introduced into the analysis for the verbal and motor dual-task conditions. It is worth mentioning that the participants in our sample were not under any gait rehabilitation program, so future studies could examine whether the predictors of gait speed under dual-task conditions change after gait training. Regarding the clinical predictors that were significant after introducing stride length and cadence into the analysis, the main predictor was the gait performance during the DYPAGS clinical test, which adds a verbal dual task to the gait assessment. This clinical test was a significant predictor in Model II in the single-task and visual dual-task gait conditions, with low scores on the test related to higher walking speeds in the aforementioned conditions [38]. The rest of the clinical predictors that were maintained in the regression models were the prescribed medication (drugs) taken (in the dual-task visual condition), H&Y stage, and the performance achieved in the FAB test (both significant predictors of gait speed during auditory dual-task). The H&Y stages are widely used in clinical practice as they allow for rapid assessment of the severity of the pathology. H&Y stages have previously been linked to gait speed [55], finding, as in our study, that the initial stages are related to a higher speed. However, we expected the H&Y stage to have a significant influence on gait speed in all of the conditions evaluated; however, in our analyses, it was only a significant predictor in the auditory dual-task condition (found both in Models I and II). This may be because the factors that affect the Parkinsonian gait can differ depending on the complexity of the environments in which the condition develops [56].

Our findings suggest that there is a need for specialized therapeutic approaches aiming to improve gait speed, since focusing only on the physical or biomechanical parameters may not address all of the factors that influence the deterioration of the Parkinsonian gait or the causes of hypokinesia [45], especially in functional contexts or multitasking environments. Similarly, sociodemographic characteristics such as the gender and academic level of patients may represent indicators of the progression of the physical performance of patients that should be taken into account. In future studies analyzing the predictors of the biomechanical performance of people with PD, all types of predictors should be taken into account, as well as studying larger samples, to verify the usefulness of the regression results generated in this study. Moreover, this study failed to include subjects with cognitive impairments or with common complications such as freezing of gait, dyskinesia, or with co-diagnostics as depression. Therefore, our findings cannot be extrapolated beyond the segment of the PD population studied. Future studies should analyze the gait speed in relation to different domains, such as the stages of the disease in which a quantifiable cognitive impairment appears.

## 5. Conclusions

In summary, our regression model based on the characteristics of the participants and clinical results explained 47% of the gait speed variability in single-task conditions. However, when dual-task conditions were assessed, it was only possible to explain between 11 and 43% of the walking speed due to the complexity of tasks that may involve other executive centers. The gender, prescribed medication (drugs) taken, and academic level, along with the FAB, DYPAGS, and PDQ-39 scores, contribute to predicting the walking speed when spatiotemporal parameters are not included in the regression model. These predictive factors highlight the multifactorial nature of the gait, which contributes to being able to condition the success of gait speed in functional or highly demanding environments.

## Figures and Tables

**Table 1:** Demographic and disease outcomes.

	Mean (SD)	Median	IQR	Shapiro-Wilk Test
Age (years)	66.72 (7.50)	67	9.25	*p* = 0.08
BMI	26.51 (4.63)	26.41	4.94	*p* = 0.55
Evolution (years)	5.78 (4.67)	4	8.50	*p* = 0.01
N° comorbidities	1.91 (1.34)	2	2	*p* = 0.01
N° drugs	4.35 (2.04)	4	3	*p* = 0.26
	**Frequency**		**(%)**	
Hoehn and Yahr scale	I:4		10	
	II:9		22.5	
	III:27		67.5	
Gender	(I) Female: 23		57.5	
	(II) Male: 17		42.5	
Academic level	I: 5 II: 3 III: 19 IV: 4 V: 9		12.5 7.5 47.5 10 22.5	

Main demographic data for Parkinson’s disease participants. Mean, standard deviation (SD), median, interquartile range (IQR), and Shapiro–Wilk test for normality assumption are shown for discrete quantitative variables. Frequency is informed by categorical outcomes. BMI: body mass index. Academic levels: I, Elementary school; II, Middle School; III, High school; IV, Technical professional training; IV, University degree studies.

**Table 2:** Clinical test and spatiotemporal outcomes.

**Clinical Test Measures**			
		**Range**	**Mean (SD)**
Minimental		25–32	29.50 (0.34)
FAB		9–29	16.25 (2.84)
TMTa (s)		23–225	54.46 (5.71)
TMTb (s)		40–315	116.53 (8.83)
PDQ39		0.51–46.40	19.68 (9)
TUG (s)		7–18	11.47 (3.01)
Tinetti		15–28	21.87 (3.27)
DYPGS		0–15	6.96 (3.73)
**Biomechanical Outcomes Measures**			
		**Range**	**Mean (SD)**
Gait velocity (m/s)	ST DTvi DTve DTa DTm	0.65–1.36 0.51–1.24 0.42–1.54 0.45–1.28 0.35–1.20	0.97 (0.19) 0.90 (0.21) 0.83 (0.22) 0.85 (0.19) 0.82 (0.21)
Stride length (m)	ST DTvi DTve DTa DTm	0.75–1.57 0.63–1.36 0.59–1.38 0.56–1.38 0.49–1.44	1.08 (0.18) 1.01 (0.18) 0.96 (0.18) 1.00 (0.18) 0.94 (0.26)
Cadence (steps/min)	ST DTvi DTve DTa DTm	91.84–137.75 68.59–192.17 72.79–193.58 79.80–198.15 65.70–186.23	110.87 (9.72) 110.52 (18.65) 106.07 (19.61) 108.58 (19.37) 108.34 (18.90)

Clinical test and spatiotemporal outcomes from participants with Parkinson’s disease. Range, mean, standard deviation (SD) are shown.

**Table 3:** Models I. Summary of Multiple Linear Regression Analysis Predicting Gait Speed from sociodemographic and clinical tests significant predictors.

Gait Condition	Predictor	Unstandardized		Standardized	CI	t	*p*
		β	SE	β			
ST R^2^ = 0.47	FAB	0.04	0.00	0.54	0 0.01, 0.05	4.21	0.00
	Sex	0.14	0.04	0.36	0.003, 0.23	2.81	0.00
	DYPGS	−0.02	0.00	−0.31	−0.02, −0.01	−2.42	0.02
DTvi R^2^ = 0.24	DYPGS	−0.02	0.00	−0.39	−0.01, −0.04	−2.65	0.01
	N° drugs	−0.03	0.01	−0.32	−0.07, −0.01	−2.17	0.03
DTve R^2^ = 0.11	Academic degree	0.06	0.02	0.33	0.01, 0.11	2.08	0.04
DTa R^2^ = 0.25	FAB	0.03	0.00	0.43	0.01, 0.04	2.89	0.00
	H&Y	−0.09	0.04	−0.33	−0.18, −0.01	−2.22	0.03
DTm R^2^ = 0.43	DPGS	−0.03	0.00	−0.46	−0.04, −0.01	−3.5	0.00
	FAB	0.03	0.00	0.36	0.01, 0.04	2.8	0.00
	PDQ39	−0.01	0.00	−0.28	−0.01, −0.01	−0.28	0.03

Gait conditions: ST, single-task; DTvi, visual dual-task; DTve, verbal dual-task; DTa, auditory dual-task; DTm, motor dual-task. Predictors: FAB, frontal assessment battery; DPGS, dynamic Parkinson gait scale; H&Y, Hoehn and Yahr stage of disease severity; PDQ39, Parkinson’s disease questionnaire-39. Statistics: SE, standard error; CI, confidence interval.

**Table 4:** Models II. Summary of Multiple Linear Regression Analysis Predicting Gait Speed to introduce the spatiotemporal parameter into model I.

Gait Condition	Predictor	Unstandardized		Standardized	CI	t	*p*
		β	SE	β			
ST R^2^ = 0.93	Stride length	0.74	0.04	0.81	0.65, 0.82	18.07	0.00
	Cadence	0.01	0.00	0.38	0.01, 0.01	8.93	0.00
	DYPGS	−0.01	0.00	−0.13	−0.01, −0.00	−2.97	0.00
DTvi R^2^ = 0.24	DYPGS	0.02	0.00	0.39	0.01, 0.04	2.65	0.01
	Nº Drugs	−0.03	0.01	−0.32	−0.07, −0.00	−2.17	0.03
DTve R^2^ = 0.94	Cadence	0.01	0.00	0.68	0.01, 0.01	17.77	0.00
	Stride length	0.73	0.04	0.66	0.64, 0.81	16.95	0.00
DTa R^2^ = 0.81	Stride length	0.61	0.07	0.65	0.45, 0.75	8.08	0.00
	Cadence	0.00	0.00	0.37	0.00, 0.01	4.86	0.00
	FAB	0.02	0.00	0.27	0.00, 0.03	3.35	0.00
	H&Y	−0.06	0.02	−0.19	−0.09, −0.01	−2.53	0.01
DTm R^2^ = 0.43	Stride length	0.62	0.03	0.88	0.55, 0.69	17.30	0.00
	Cadence	0.01	0.00	0.75	0.00, 0.01	14.86	0.00

Gait conditions: ST, single task; DTvi, visual dual task; DTve, verbal dual task; DTa, auditory dual-task; DTm, motor dual task. Predictors: DPGS, dynamic Parkinson gait scale; FAB, frontal assessment battery; H&Y, Hoehn and Yahr stage of disease severity. Statistics: SE, standard error; CI, confidence interval.

## Data Availability

Data available on request due to privacy and ethical restrictions.

## References

[1] Jankovic J. (2008). Parkinson’s Disease: Clinical Features and Diagnosis. J. Neurol. Neurosurg. Psychiatry.

[2] Soh S.-E., Morris M.E., McGinley J.L. (2011). Determinants of Health-Related Quality of Life in Parkinson’s Disease: A Systematic Review. Parkinsonism Relat. Disord..

[3] Tan D., Danoudis M., McGinley J., Morris M.E. (2012). Relationships between Motor Aspects of Gait Impairments and Activity Limitations in People with Parkinson’s Disease: A Systematic Review. Parkinsonism Relat. Disord..

[4] Rahman S., Griffin H.J., Quinn N.P., Jahanshahi M. (2008). Quality of Life in Parkinson’s Disease: The Relative Importance of the Symptoms. Mov. Disord..

[5] Muslimovic D., Post B., Speelman J.D., Schmand B., de Haan R.J. (2008). CARPA Study Group Determinants of Disability and Quality of Life in Mild to Moderate Parkinson Disease. Neurology.

[6] Kelly V.E., Eusterbrock A.J., Shumway-Cook A. (2012). A Review of Dual-Task Walking Deficits in People with Parkinson’s Disease: Motor and Cognitive Contributions, Mechanisms, and Clinical Implications. Park. Dis..

[7] Sofuwa O., Nieuwboer A., Desloovere K., Willems A.-M., Chavret F., Jonkers I. (2005). Quantitative Gait Analysis in Parkinson’s Disease: Comparison With a Healthy Control Group. Arch. Phys. Med. Rehabil..

[8] San Martín Valenzuela C., Dueñas Moscardó L., López-Pascual J., Serra-Añó P., Tomás J.M. (2020). Interference of Functional Dual-Tasks on Gait in Untrained People with Parkinson’s Disease and Healthy Controls: A Cross-Sectional Study. BMC Musculoskelet. Disord..

[9] Fritz S., Lusardi M. (2009). White Paper: “Walking Speed: The Sixth Vital Sign”. J. Geriatr. Phys. Ther..

[10] Castell M.-V., Sánchez M., Julián R., Queipo R., Martín S., Otero Á. (2013). Frailty Prevalence and Slow Walking Speed in Persons Age 65 and Older: Implications for Primary Care. BMC Fam. Pract..

[11] Middleton A., Fritz S.L., Lusardi M. (2015). Walking Speed: The Functional Vital Sign. J. Aging Phys. Act..

[12] Paker N., Bugdayci D., Goksenoglu G., Demircioğlu D.T., Kesiktas N., Ince N. (2015). Gait Speed and Related Factors in Parkinson’s Disease. J. Phys. Ther. Sci..

[13] Morris R., Lord S., Lawson R.A., Coleman S., Galna B., Duncan G.W., Khoo T.K., Yarnall A.J., Burn D.J., Rochester L. (2017). Gait Rather Than Cognition Predicts Decline in Specific Cognitive Domains in Early Parkinson’s Disease. J. Gerontol. Ser. A.

[14] Pieruccini-Faria F., Black S.E., Masellis M., Smith E.E., Almeida Q.J., Li K.Z.H., Bherer L., Camicioli R., Montero-Odasso M. (2021). Gait Variability across Neurodegenerative and Cognitive Disorders: Results from the Canadian Consortium of Neurodegeneration in Aging (CCNA) and the Gait and Brain Study. Alzheimers Dement..

[15] Toots A.T.M., Taylor M.E., Lord S.R., Close J.C.T. (2019). Associations Between Gait Speed and Cognitive Domains in Older People with Cognitive Impairment. J. Alzheimers Dis..

[16] Seo M., Won C.W., Kim S., Yoo J.H., Kim Y.H., Kim B.S. (2020). The Association of Gait Speed and Frontal Lobe among Various Cognitive Domains: The Korean Frailty and Aging Cohort Study (KFACS). J. Nutr. Health Aging.

[17] Ebersbach G., Moreau C., Gandor F., Defebvre L., Devos D. (2013). Clinical Syndromes: Parkinsonian Gait. Mov. Disord..

[18] Bortone I., Sardone R., Lampignano L., Castellana F., Zupo R., Lozupone M., Moretti B., Giannelli G., Panza F. (2021). How Gait Influences Frailty Models and Health-related Outcomes in Clinical-based and Population-based Studies: A Systematic Review. J. Cachexia Sarcopenia Muscle.

[19] Yates T., Razieh C., Zaccardi F., Rowlands A.V., Seidu S., Davies M.J., Khunti K. (2021). Obesity, Walking Pace and Risk of Severe COVID-19 and Mortality: Analysis of UK Biobank. Int. J. Obes..

[20] Beauchet O., Berrut G. (2006). Gait and dual-task: Definition, interest, and perspectives in the elderly. Psychol. Neuropsychiatr. Vieil..

[21] Kandel E.R., Schwartz J.H., Jessell T.M., Siegelbaum S., Hudspeth A.J., Mack S. (2013). Principles of Neural Science.

[22] Eisinger R.S., Cernera S., Gittis A., Gunduz A., Okun M.S. (2019). A Review of Basal Ganglia Circuits and Physiology: Application to Deep Brain Stimulation. Parkinsonism Relat. Disord..

[23] Rochester L., Nieuwboer A., Baker K., Hetherington V., Willems A.-M., Kwakkel G., Van Wegen E., Lim I., Jones D. (2008). Walking Speed during Single and Dual Tasks in Parkinson’s Disease: Which Characteristics Are Important?. Mov. Disord..

[24] San Martín Valenzuela C., Moscardó L.D., López-Pascual J., Serra-Añó P., Tomás J.M. (2020). Effects of Dual-Task Group Training on Gait, Cognitive Executive Function, and Quality of Life in People With Parkinson Disease: Results of Randomized Controlled DUALGAIT Trial. Arch. Phys. Med. Rehabil..

[25] Isella V., Mapelli C., Morielli N., De Gaspari D., Siri C., Pezzoli G., Antonini A., Poletti M., Bonuccelli U., Picchi L. (2013). Validity and Metric of MiniMental Parkinson and MiniMental State Examination in Parkinson’s Disease. Neurol. Sci..

[26] Morris M.E., McGinley J., Huxham F., Collier J., Iansek R. (1999). Constraints on the Kinetic, Kinematic and Spatiotemporal Parameters of Gait in Parkinson’s Disease. Hum. Mov. Sci..

[27] Brauer S.G., Morris M.E. (2010). Can People with Parkinson’s Disease Improve Dual Tasking When Walking?. Gait Posture.

[28] Peto V., Jenkinson C., Fitzpatrick R., Greenhall R. (1995). The Development and Validation of a Short Measure of Functioning and Well Being for Individuals with Parkinson’s Disease. Qual. Life Res. Int. J. Qual. Life Asp. Treat. Care Rehabil..

[29] Moreira R.C., Zonta M.B., de Araújo A.P.S., Israel V.L., Teive H.A.G., Moreira R.C., Zonta M.B., de Araújo A.P.S., Israel V.L., Teive H.A.G. (2017). Quality of Life in Parkinson’s Disease Patients: Progression Markers of Mild to Moderate Stages. Arq. Neuropsiquiatr..

[30] Martínez M., Frades B., Jiménez J., Pondal M., López L., Vela L., Vázquez A., Del V.F., Diví M., Fabregat N. (1999). The PDQ-39 Spanish Version: Reliability and Correlation with the Short- Form Health Survey (SF-36). Neurologia.

[31] Pomares M.H., Gran D.V., Pérez A.S., Gómez P.P., Muñoz E.M.N., del Cantero M.C.T. (2021). Adaptation of the Spanish Version of the Frontal Assessment Battery for Detection of Executive Dysfunction. Med. Clin..

[32] Dubois B., Slachevsky A., Litvan I., Pillon B. (2000). The FAB: A Frontal Assessment Battery at Bedside. Neurology.

[33] Romann A.J., Dornelles S., de Maineri N.L., de Rieder C.R.M., Olchik M.R. (2012). Cognitive Assessment Instruments in Parkinson’s Disease Patients Undergoing Deep Brain Stimulation. Dement. Neuropsychol..

[34] Arango-Lasprilla J.C., Rivera D., Aguayo A., Rodríguez W., Garza M.T., Saracho C.P., Rodríguez-Agudelo Y., Aliaga A., Weiler G., Luna M. (2015). Trail Making Test: Normative Data for the Latin American Spanish Speaking Adult Population. NeuroRehabilitation.

[35] Fernández A.L., Marino J.C., Alderete A.M. (2002). Estandarización y Validez Conceptual Del Test Del Trazo En Una Muestra de Adultos Argentinos. Rev. Neurol. Argent..

[36] Do Silva R.N., Afonso S.V., Felipe L.R., Oliveira R.A., Martins L.J.P., de Souza L.A.P.S. (2021). Dual-Task Intervention Based on Trail Making Test: Effects on Parkinson’s Disease. J. Bodyw. Mov. Ther..

[37] Bezdicek O., Stepankova H., Axelrod B.N., Nikolai T., Sulc Z., Jech R., Růžička E., Kopecek M. (2017). Clinimetric Validity of the Trail Making Test Czech Version in Parkinson’s Disease and Normative Data for Older Adults. Clin. Neuropsychol..

[38] Crémers J., Phan Ba R., Delvaux V., Garraux G. (2012). Construction and Validation of the Dynamic Parkinson Gait Scale (DYPAGS). Parkinsonism Relat. Disord..

[39] Tinetti M.E. (1986). Performance-Oriented Assessment of Mobility Problems in Elderly Patients. J. Am. Geriatr. Soc..

[40] Kegelmeyer D.A., Kloos A.D., Thomas K.M., Kostyk S.K. (2007). Reliability and Validity of the Tinetti Mobility Test for Individuals with Parkinson Disease. Phys. Ther..

[41] Podsiadlo D., Richardson S. (1991). The Timed “Up & Go”: A Test of Basic Functional Mobility for Frail Elderly Persons. J. Am. Geriatr. Soc..

[42] Palmerini L., Mellone S., Avanzolini G., Valzania F., Chiari L. (2013). Quantification of Motor Impairment in Parkinson’s Disease Using an Instrumented Timed up and Go Test. IEEE Trans. Neural Syst. Rehabil. Eng..

[43] Vereeck L., Wuyts F., Truijen S., de Heyning P.V. (2008). Clinical Assessment of Balance: Normative Data, and Gender and Age Effects. Int. J. Audiol..

[44] Mollinedo I., Ma Cancela J. (2020). Evaluation of the Psychometric Properties and Clinical Applications of the Timed Up and Go Test in Parkinson Disease: A Systematic Review. J. Exerc. Rehabil..

[45] Shearin S., Medley A., Trudelle-Jackson E., Swank C., Querry R. (2021). Differences in Predictors for Gait Speed and Gait Endurance in Parkinson’s Disease. Gait Posture.

[46] Tabachnick B.G., Fidell L.S. (2018). Using Multivariate Statistics.

[47] Wu T., Hallett M. (2008). Neural Correlates of Dual Task Performance in Patients with Parkinson’s Disease. J. Neurol. Neurosurg. Psychiatry.

[48] Shumway-Cook A., Woollacott M.H. (2012). Motor Control. Translating Research into Clinical Practice.

[49] Kyrönlahti S.M., Stenholm S., Raitanen J., Neupane S., Koskinen S., Tiainen K. (2021). Educational Differences in Decline in Maximum Gait Speed in Older Adults Over an 11-Year Follow-Up. J. Gerontol. A Biol. Sci. Med. Sci..

[50] Deguchi M., Nishida K., Enokiya T., Ooi K. (2019). Risk Factor Analysis of the Decrease in Gait Speed among Japanese Older Outpatients with Polypharmacy. J. Pharm. Health Care Sci..

[51] Naples J.G., Marcum Z.A., Perera S., Newman A.B., Greenspan S.L., Gray S.L., Bauer D.C., Simonsick E.M., Shorr R.I., Hanlon J.T. (2016). Impact of Drug-Drug and Drug-Disease Interactions on Gait Speed in Community-Dwelling Older Adults. Drugs Aging.

[52] Ebersbach G., Sojer M., Valldeoriola F., Wissel J., Müller J., Tolosa E., Poewe W. (1999). Comparative Analysis of Gait in Parkinson’s Disease, Cerebellar Ataxia and Subcortical Arteriosclerotic Encephalopathy. Brain J. Neurol..

[53] Morris M.E., Iansek R., Matyas T.A., Summers J.J. (1994). The Pathogenesis of Gait Hypokinesia in Parkinson’s Disease. Brain J. Neurol..

[54] Morris M.E., Iansek R., Matyas T.A., Summers J.J. (1996). Stride Length Regulation in Parkinson’s Disease. Normalization Strategies and Underlying Mechanisms. Brain J. Neurol..

[55] Vila M.H., Pérez R., Mollinedo I., Cancela J.M. (2021). Analysis of Gait for Disease Stage in Patients with Parkinson’s Disease. Int. J. Environ. Res. Public. Health.

[56] Nohelova D., Bizovska L., Vuillerme N., Svoboda Z. (2021). Gait Variability and Complexity during Single and Dual-Task Walking on Different Surfaces in Outdoor Environment. Sensors.

